# Exome Sequencing Identifies Genetic Variants Associated with Circulating Lipid Levels in Mexican Americans: The Insulin Resistance Atherosclerosis Family Study (IRASFS)

**DOI:** 10.1038/s41598-018-23727-2

**Published:** 2018-04-04

**Authors:** Chuan Gao, Keri L. Tabb, Latchezar M. Dimitrov, Kent D. Taylor, Nan Wang, Xiuqing Guo, Jirong Long, Jerome I. Rotter, Richard M. Watanabe, Joanne E. Curran, John Blangero, Carl D. Langefeld, Donald W. Bowden, Nicholette D. Palmer

**Affiliations:** 1 Molecular Genetics and Genomics Program, Winston-Salem, NC USA; 2 Center for Genomics and Personalized Medicine Research, Winston-Salem, NC USA; 3 Department of Biochemistry, Winston-Salem, NC USA; 4 Department of Biostatistical Sciences, Wake Forest School of Medicine, Winston-Salem, NC USA; 5 Institute for Translational Genomics and Population Sciences and Department of Pediatrics, Los Angeles Biomedical Research Institute at Harbor-UCLA Medical Center, Torrance, CA USA; 6 Department of Preventive Medicine and Physiology and Biophysics, University of Southern California Keck School of Medicine, Los Angeles, CA USA; 7 Department of Medicine and Vanderbilt Epidemiology Center Vanderbilt-Ingram Cancer Center, Vanderbilt University School of Medicine, Nashville, TN USA; 8 South Texas Diabetes and Obesity Institute, University of Texas Rio Grande Valley School of Medicine, Brownsville, TX USA

## Abstract

Genome-wide association studies have identified numerous variants associated with lipid levels; yet, the majority are located in non-coding regions with unclear mechanisms. In the Insulin Resistance Atherosclerosis Family Study (IRASFS), heritability estimates suggest a strong genetic basis: low-density lipoprotein (LDL, h^2^ = 0.50), high-density lipoprotein (HDL, h^2^ = 0.57), total cholesterol (TC, h^2^ = 0.53), and triglyceride (TG, h^2^ = 0.42) levels. Exome sequencing of 1,205 Mexican Americans (90 pedigrees) from the IRASFS identified 548,889 variants and association and linkage analyses with lipid levels were performed. One genome-wide significant signal was detected in *APOA5* with TG (rs651821, P_TG_ = 3.67 × 10^−10^, LOD_TG_ = 2.36, MAF = 14.2%). In addition, two correlated SNPs (r^2^ = 1.0) rs189547099 (P_TG_ = 6.31 × 10^−08^, LOD_TG_ = 3.13, MAF = 0.50%) and chr4:157997598 (P_TG_ = 6.31 × 10^−08^, LOD_TG_ = 3.13, MAF = 0.50%) reached exome-wide significance (P < 9.11 × 10^−08^). rs189547099 is an intronic SNP in *FNIP2* and SNP chr4:157997598 is intronic in *GLRB*. Linkage analysis revealed 46 SNPs with a LOD > 3 with the strongest signal at rs1141070 (LOD_LDL_ = 4.30, P_LDL_ = 0.33, MAF = 21.6%) in *DFFB*. A total of 53 nominally associated variants (P < 5.00 × 10^−05^, MAF ≥ 1.0%) were selected for replication in six Mexican-American cohorts (N = 3,280). The strongest signal observed was a synonymous variant (rs1160983, P_LDL_ = 4.44 × 10^−17^, MAF = 2.7%) in *TOMM40*. Beyond primary findings, previously reported lipid loci were fine-mapped using exome sequencing in IRASFS. These results support that exome sequencing complements and extends insights into the genetics of lipid levels.

## Introduction

Cardiovascular disease (CVD) is the leading cause of death worldwide^1^. In the United States, CVD accounts for more deaths than any other major cause and, on average, 2,200 Americans die of CVD each day^2^. While the exact mechanism of disease remains unclear, lipid concentrations are well-accepted as a major risk factor as well as clinical indicators for CVD. Genetic studies have suggested a strong heritability for circulating lipid levels, i.e. total cholesterol (TC), LDL cholesterol (LDL), HDL cholesterol (HDL), and triglycerides (TG). Based on a European twin-pairs study, it is estimated that circulating lipid heritability ranges from 0.58 to 0.66 (h^2^_HDL_ = 0.61, h^2^_LDL_ = 0.59, h^2^_TC_ = 0.58, h^2^_TG_ = 0.66)^3^.

Given the public health relevance as well as the strong genetic component, numerous genome-wide association studies (GWAS) have been performed to investigate the genetic architecture of circulating lipid levels. The most recent Global Lipid Genetics Consortium (GLGC) analyzed 188,577 individuals from four ethnicities (Europeans, East Asians, South Asians, and Africans) and identified 157 loci associated with plasma lipid traits^4^. While well-powered, these efforts have largely overlooked the fastest growing US minority population, Hispanic Americans. Compared to non-Hispanic whites, Hispanics suffer an even higher risk for CVD, i.e. 32.1% versus 23.8%^5,6^. Until now, the largest lipid GWAS in Hispanics was performed by Below *et al*. in 2015 with 4,383 Mexican ancestry individuals. However, all genome-wide significant regions identified in the Mexican meta-analysis were previously identified^4,7–9^.

GWAS were designed with a focus on common variants, partly supported by the “common disease, common variant” hypothesis^10^. However, despite the large number of genetic signals identified by GWAS, over 80% fall outside of protein coding regions, which complicates causal inference^10^. Among genes with evidence of association, only a small proportion of the variance is explained, providing limited information for disease risk prediction^4,11^. Sequencing of the exome, rather than the entire human genome, has been shown to be an efficient strategy to search for novel variants with a clear biological mechanism^12^. Previous exome sequencing studies have identified multiple rare variants associated with CVD in non-Hispanic populations^13,14^.

To search for functional coding variants regulating circulating lipid levels in a Mexican-ancestry population, whole exome sequencing was performed in 1,205 Mexican Americans from the Insulin Resistance Atherosclerosis Family Study (IRASFS). Association and family-based linkage analyses were performed for 548,889 variants. We hypothesized that a family cohort would have increased power to detect rare variants due to their transmission in multigenerational pedigrees. With the complimentary approaches of linkage and association, exome sequencing has the potential to identify ethnic-specific variants regulating lipid levels.

## Results

A total of 1,205 individuals were included in association and linkage analyses. Characteristics of the study individuals are shown in Table 1. Overall, individuals were predominantly female (59%) and were overweight with an average BMI of 28.9 kg/m^2^. Since the recruitment was based on family size rather than diagnosis, e.g. CVD was not required for participation, the participants were metabolically normal, with an average HDL (43.61 mg/dl), LDL (109.41 mg/dl), TC (178.06 mg/dl), and TG (124.80 mg/dl) within desirable or near-desirable ranges^15^. According to the National Cholesterol Education Program (NCEP)^15^, 183 individuals (15%) within the study had undesirable high TG levels (TG > 150 mg/dl), 121 (10%) individuals had an LDL level greater than 160 mg/dl, 521 (43%) individuals had an HDL level less than 40 mg/dl, and 290 (24%) individuals had a TC level greater than 200 mg/dl.

**Table 1:** Demographic information of the study individuals.

	Discovery	Replication					
	IRASFS	IRAS	TRIPOD	BetaGene	HTN-IR	MACAD	NIDDM-Athero
n	1205	181	125	1218	763	749	244
Female (%)	58.5	41.1	100.0	27.5	41.1	42.6	38.9
Age (years)^b^	42.7 ± 14.5	54.1 ± 8.2	34.9 ± 6.4	34.7 ± 7.9	39.3 ± 15.1	34.7 ± 9.1	38.1 ± 14.9
Body Mass Index (BMI; kg/m^2^)	28.9 ± 6.2	28.2 ± 5.1	30.8 ± 5.6	29.6 ± 6.1	29.3 ± 5.7	29.0 ± 5.2	29.1 ± 6.3
High-density Lipoprotein (HDL; mg/dl)	43.61 ± 12.86	43.18 ± 14.63	37.39 ± 9.52	46.89 ± 11.08	48.13 ± 13.24	46.14 ± 12.12	47.5 ± 11.7
Low-density Lipoprotein (LDL; mg/dl)	109.41 ± 31.04	140.04 ± 36.75	109.01 ± 27.30	102.80 ± 28.65	106.03 ± 31.38	108.23 ± 31.3	106.63 ± 32.01
Total Cholesterol (TC; mg/dl)	178.06 ± 37.45	210.90 ± 43.74	173.15 ± 32.59	172.52 ± 33.25	179.01 ± 35.41	180.53 ± 36.43	181.47 ± 39.23
Triglycerides (TG; mg/dl)	124.80 ± 84.00	157.24 ± 99.21	133.79 ± 78.91	114.12 ± 90.27	124.23 ± 78.96	137.25 ± 102.82	145.19 ± 166

Heritability analysis in IRASFS suggested a strong genetic component for lipid levels. HDL had the strongest heritability (h^2^_HDL_ = 0.57) with 17.2% of the variance explained by covariates (age, sex, center, BMI; P = 5.87 × 10^−26^), the heritability of TC was 0.53 with 10.3% of the variance explained by covariates (P = 3.12 × 10^−23^), the heritability of LDL was 0.50 with 7.3% of the variance explained by covariates (P = 1.22 × 10^−21^), and TG had the lowest heritability (h^2^_TG_ = 0.42) with 15.9% of the variance explained by covariates (P = 5.37 × 10^−14^) (Table S1).

From exome sequencing data, a total of 548,889 variants were successfully analyzed for association and linkage. Among them, 30.0% (164,591) were extremely rare variants with only one or two observations and 82.5% (452,807) were low frequency variants as defined by a MAF < 5% (Figure S1); 6.8% (N = 37,157) of the variants were insertions/deletions; 33.1% (N = 182,020) and 15% (N = 83,874) of the variants marked a non-synonymous and synonymous amino acid change in coding genes, respectively.

**Figure 1:**
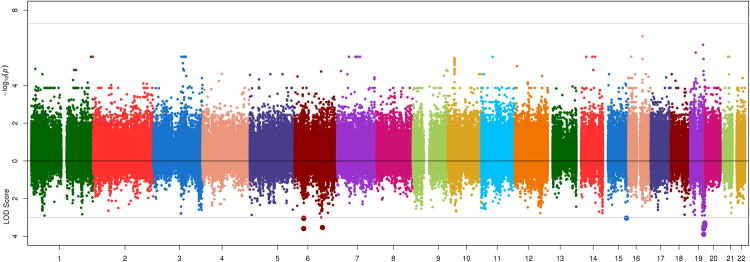
Opposed plots for association and linkage analysis of HDL. Association results are plotted on the positive y-axis. The line at –log_10_(PVAL) = 7.30 represents genome-wide significance (P = 5.00 × 10^−08^). The linkage results are plotted on the negative y-axis. The line at LOD = 3 represents a significant linkage signal.

**Figure 2:**
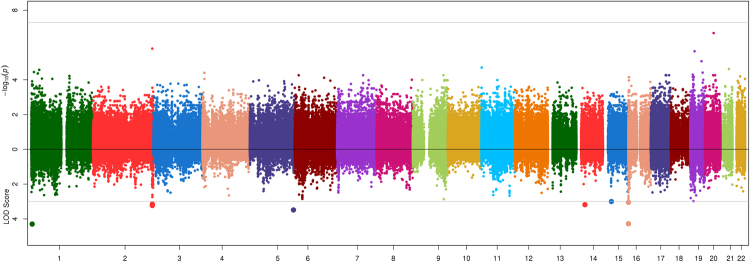
Opposed plots for association and linkage analysis of LDL. Association results are plotted on the positive y-axis. The line at –log_10_(PVAL) = 7.30 represents genome-wide significance (P = 5.00 × 10^−08^). The linkage results are plotted on the negative y-axis. The line at LOD = 3 represents a significant linkage signal.

**Figure 3:**
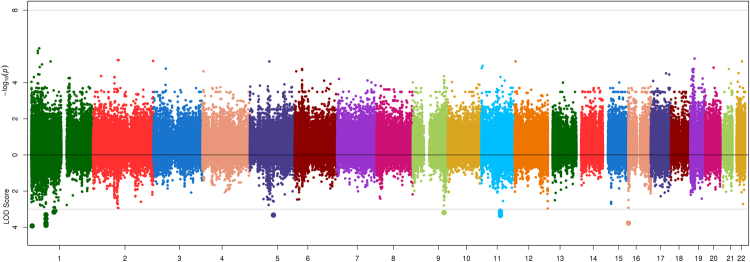
Opposed plots for association and linkage analysis of TC. Association results are plotted on the positive y-axis. The line at –log_10_(PVAL) = 7.30 represents genome-wide significance (P = 5.00 × 10^−08^). The linkage results are plotted on the negative y-axis. The line at LOD = 3 represents a significant linkage signal.

**Figure 4:**
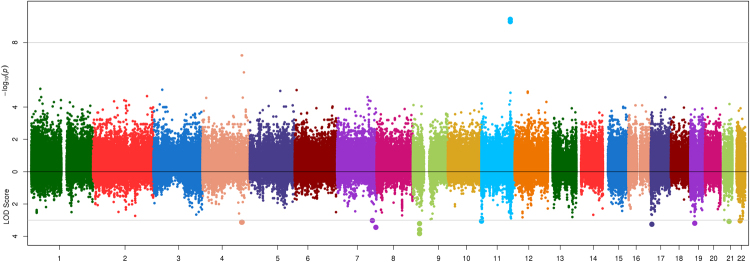
Opposed plots for association and linkage analysis of TG. Association results are plotted on the positive y-axis. The line at –log_10_(PVAL) = 7.30 represents genome-wide significance (P = 5.00 × 10^−08^). The linkage results are plotted on the negative y-axis. The line at LOD = 3 represents a significant linkage signal.

Association and linkage results are shown in Figs 1–4. The strongest evidence of association attaining genome-wide significance (P < 5.00 × 10^−08^) was observed at the apolipoprotein A-V gene (*APOA5*) on chromosome 11 with two highly correlated SNPs (r^2^ = 0.92) rs651821 (P = 3.67 × 10^−10^, LOD = 2.36, MAF = 14%) and rs2072560 (P = 5.14 × 10^−10^, LOD = 2.05, MAF = 13%) and TG (Fig. 4; Table 2). Conditional analysis on one variant was able to abolish the signal limiting the ability to statistically implicate a functional variant. In addition, both SNPs were also nominally associated with HDL (P_rs651821_ = 2.63 × 10^−3^; P_rs2072560_ = 9.42 × 10^−3^), while no signal was detected for LDL (P > 0.90) or TC (P > 0.18). On average, individuals had 23% more TG for each risk allele carried at rs651821 (TT: 117.59 mg/dl, TC: 142.62 mg/dl, CC: 174.41 mg/dl). Nominally associated and linked signals are provided in Table S2.

**Table 2:** Top association (P < 9.11 × 10^−08^) and linkage signals (LOD > 4).

SNP	Chr:Pos (hg19)	Gene	Annotation	Alleles^a^	RAF^b^	P_HDL_	LOD_HDL_	P_LDL_	LOG_LDL_	P_TC_	LOD_TC_	P_TG_	LOD_TG_
rs1141070	1:3786189	*DFFB*	coding-synon	A/G	0.22	0.98	0.01	0.33	**4.30**	0.74	**3.93**	0.87	0.27
chr4:157997598	4:157997598	*GLRB*	intron	T/C	0.0050	2.88E-04	1.87	0.71	0	0.24	0	**6.31E-08**	**3.13**
rs189547099	4:159814881	*C4orf45*	intron	C/G	0.0050	2.88E-04	1.87	0.71	0	0.24	0	**6.31E-08**	**3.13**
rs2072560	11:116661826	*APOA5*	intron	T/C	0.13	2.03E-03	0.32	0.87	0	0.014	0.010	**5.14E-10**	2.06
rs651821	11:116662579	*APOA5*	intron	C/T	0.14	8.93E-04	0.30	0.99	0	0.018	0	**3.67E-10**	2.36
rs11648905	16:425298	*TMEM8A*	intron	T/G	0.41	0.74	0	0.78	**4.28**	0.84	**3.77**	0.18	0.82

^a^Reference (minor)/Other allele; ^b^Reference allele frequency based on the entire population.

Additional evidence of association which attained exome-wide significance (P < 9.11 × 10^−08^; based on a conservative Bonferroni correction for 548,889 variants) included two correlated SNPs (r^2^ = 1.0) on chromosome 4: rs189547099 (P_TG_ = 6.31 × 10^−08^, LOD_TG_ = 3.13, MAF = 0.5%) and chr4:157997598 (P_TG_ = 6.31 × 10^−08^, LOD_TG_ = 3.13, MAF = 0.5%) (Table 2). Notably, these variants were both significantly associated and linked with TG. SNP rs189547099 is an intronic SNP located in the folliculin interacting protein 2 gene (*FNIP2*) and the chromosome 4 open reading frame 45 (*C4orf25*). SNP chr4:157997598 is located 1,817 kb upstream of SNP rs189547099 in the first intron of the glycine receptor beta gene (*GLRB*). On average, risk allele (T) heterozygous carriers for chr4:157997598 had 2.9 times TG compared to non-carriers (CC: 124 mg/dl vs. TC: 358 mg/dl). No risk allele homozygotes were found. Burden testing failed to identify additional genes significantly associated with lipid phenotypes after correction for the number of test performed, i.e. P < 7.23 × 10^−06^, 0.05/139,173; Table S3.

Two-point linkage analysis was performed for 548,889 variants. Of these, two SNPs had a LOD score greater than 4 (Table 2), 46 SNPs had a LOD score greater than 3 (Table S4). Among these, 14 variants were significantly linked (LOD > 3) with TC, 13 variants were significantly linked with HDL, 13 variants were significantly linked with TG, and 8 variants were significantly linked with LDL with two variants overlapping between TC and LDL. The strongest linkage signal was observed at SNP rs1141070 (LOD_LDL_ = 4.30 LOD_TC_ = 3.93, MAF = 22%) with LDL and TC levels. rs1141070 is located in exon 5 of the DNA fragmentation factor gene (*DFFB*) on chromosome 1 and marks a synonymous amino acid substitution. In addition, SNP rs11648905 (LOD_LDL_ = 4.28, LOD_TC_ = 3.77, MAF = 41%) was strongly linked with LDL and TC levels. This variant is an intronic SNP located between exon 7 and 8 of the transmembrane protein 8 A gene (*TMEM8A*). No significant association signals were observed for the two linked signals: rs1141070, P_LDL_ = 0.33, P_TC_ = 0.74; rs11648905, P_LDL_ = 0.78, P_TC_ = 0.84 (Table 2).

### Meta-analysis

Meta-analysis with six additional independent cohorts was computed for the 53 selected SNPs. Overall, six variants reached genome-wide significance after meta-analysis (Table 3). The strongest signal was observed at rs1160983 (P_LDL_ = 4.44 × 10^−17^, MAF = 2.7%) with LDL. rs1160983 is a synonymous coding variant in the translocase of outer mitochondrial membrane 40 gene (*TOMM40*). The two *APOA5* variants, which attained genome-wide significance in IRASFS, were also successfully replicated (meta-analysis p-values: rs2072560, P_TG_ = 5.67 × 10^−16^; rs651821, P_TG_ = 2.66 × 10^−15^) with a consistent direction of effect across all cohorts. In addition, strong meta-analysis signals were detected for *APOA1* (rs2070665, P_TG_ = 7.03 × 10^−09^) and *CETP* (rs1532625, P_HDL_ = 7.72 × 10^−14^, rs11076176, P_HDL_ = 2.15 × 10^−08^) with TG and HDL, respectively. SNP rs72685601 was selected as the proxy SNP (r^2^ = 0.59) for the two variants that reached exome-wide significance (chr4:157997598, rs189547099). It was nominally associated with TG (P_TG_ = 3.69 × 10^−03^) with consistent direction of effect across six of the seven cohorts. A complete list of meta-analysis results can be found in Table S5.

**Table 3:** Top association signals (P < 5 × 10^−8^) from meta-analysis.

SNP	Chr:Pos (hg19)	RAF^a^	Trait	Gene	Annotation	IRASFS		Meta-analysis	
						N	P	N	P
rs2072560	11:116661826	0.13	TG	*APOA5*	intron	1205	5.14E-10	4241	5.67E-16
rs651821	11:116662579	0.14	TG	*APOA5*	intron	1205	3.67E-10	4241	2.66E-15
rs2070665	11:116707684	0.17	TG	*APOA1*	intron	1205	4.10E-05	4241	7.03E-09
rs1532625	16:57005301	0.38	HDL	*CETP*	intron	1205	2.46E-07	4235	7.72E-14
rs11076176	16:57007446	0.28	HDL	*CETP*	intron	1205	3.87E-06	4235	2.15E-08
rs1160983	19:45397229	0.027	LDL	*TOMM40*	synonymous	1205	8.61E-06	4177	4.44E-17

^a^Reference allele frequency based on the IRASFS cohort.

### Previously identified lipid loci

Fine-mapping of the previously identified 157 loci +/−100 kb resulted in a total of 14,232 exome sequencing variants which were analyzed for association and linkage in IRASFS. In addition, conditional analyses based on the locus-specific GLGC index SNP were performed (Table S6). For association, 1,231 of the variants were identified with a P-value less than 0.05 with at least one of the reported lipid traits. Among the 1,231 significant variants, 646 remained to be significant (P < 0.05) after conditional analysis based on the locus-specific index SNP. The strongest association signal was observed at rs651821 (P_TG_ = 3.67 × 10^−10^, LOD_TG_ = 2.36) in *APOA5*. After conditioning on SNP rs964184, it remained nominally associated and linked with TG (P_TG|rs964184_ = 1.76 × 10^−04^, LOD_TG|rs964184_ = 0.99). In addition, SNP rs1532625 also survived the stringent Bonferroni correction (P < 3.51 × 10^−06^ for 14,232 variants): P_HDL_ = 2.46 × 10^−07^, LOD_HDL_ = 1.42. This SNP is an intronic variant located in the cholesteryl ester transfer protein gene (*CETP*). However, conditional analysis with the index SNP rs3764261 totally abolished the signal (P_HDL|rs3764261_ = 0.61, LOD_HDL|rs3764261_ = 0.02). For linkage analysis, 14 and 127 SNPs reached a LOD score greater than two and one, respectively, for at least one reported lipid trait. Among them, one (rs1134760, LOD_HDL|rs16942887_ = 2.46) and 28 (LOD > 1) remained to be linked after conditional analysis, respectively.

## Discussion

An individual’s lipid profile represents a well-accepted major risk factor and clinical indicator of CVD. In this study, heritability estimates for four lipid phenotypes were reported in Mexican Americans from IRASFS and demonstrated a strong genetic component. Subsequently, exome-wide association analysis was performed using exome sequencing data derived from 1,205 Mexican Americans from IRASFS. Multiple significant association signals were identified and top signals were evaluated in six additional independent cohorts (n = 3,280). As a complementary approach to association, linkage analysis was performed to identify rare variant signals. In addition, 157 previously identified lipid loci were fine-mapped using exome sequencing data in IRASFS.

Strong association and suggestive linkage signals were observed with two SNPs in *APOA5*: rs651821 (P_TG_ = 3.67 × 10^−10^, LOD_TG_ = 2.36, MAF = 14%) and rs2072560 (P_TG_ = 5.14 × 10^−10^, LOD_TG_ = 2.05, MAF = 13%). *APOA5* encodes an apoliporotein that plays an important role in regulating plasma triglyceride levels, which is a strong risk factor for CVD^16^. This gene is located within the apolipoprotein gene cluster on chromosome 11q23.3, which contains multiple lipid-related genes including *APOA1*, *APOA3*, *APOA4*, *APOA5*, and *PCSK7*. Multiple strong association signals have been identified in the region with HDL, TG, and TC^4,9,17^. In 2015, Do *et al*.^14^ described a large exome sequencing study in 9,793 European and African Americans and identified strong associations between *APOA5* functional variants and myocardial infraction (MI). A recent Hispanic GWAS of lipid phenotypes identified SNP rs964184, 359 bases downstream of zinc finger protein 259 (*ZNF259*) and 11 kb downstream of *APOA5*, as significantly associated with TG^7^. In addition, Parra *et al*. presented robust association for rs964184 and no comparable signals were identified in the 5’ UTR for *APOA5*^18^. In IRASFS, SNP rs964184 was also associated with TG (P = 4.79 × 10^−07^). However, including SNP rs964184 as a covariant failed to completely abolish the genetic signal of rs651821 (P_before_ = 3.67 × 10^−10^, P_after_ = 1.76 × 10^−−04^), suggesting that the two signals were likely independent (r^2^ = 0.37) (Figure S2).

In IRASFS, two common variants (rs651821, rs2072560) within *APOA5* were identified with strong association signals with TG in a Mexican-American family cohort. While SNP rs651821 and rs2072560 have been previously identified to be strongly associated with TG in multiple ethnicities (Europeans, East Asians, and North Africans)^19–23^, this is the first reported evidence in a Mexican-ancestry population. SNP rs651821 is a 5′-UTR variant that is three bases upstream of the coding exon. Worth mentioning, rs651821 is also located in the binding site of transcription factor POLR2A as suggested in HepG2 cells by ENCODE^24^. Previous expression quantitative trait loci (eQTL) studies revealed associations between rs651821 and transgelin (*TAGLN*) gene expression levels^25^ yet no *APOA5* expression regulation effect was found. SNP rs2072560 is located intronically between exons 3 and 4 of *APOA5*. Interestingly, rs2072560 is also a missense variant of an alternative transcript of *APOA5* (NM_001166598) which contains two exons. This variant marks a glutamic acid to glycine amino acid change (E66G) in exon 2 (Figure S5). To further explore the potential function of the alternative transcript, four ENCODE primary hepatocyte RNA sequencing experiments were analyzed and plotted using the UCSC genome browser^24,26^. However, no RNA sequencing evidence was found to support existence or function of the alternative transcript (Figure S5). Taken together, strong association, linkage, and replication signals were identified for the two *APOA5* SNPs with TG in Mexican Americans. While not enough biological evidence was found to support their causality, the results refined the scope of the *APOA5* association signals and provided information for future efforts to locate the causal variant in the region.

While not attaining strict genome-wide significance, two correlated SNPs (r^2^ = 1.0) rs189547099 (P_TG_ = 6.31 × 10^−08^, LOD_TG_ = 3.13, MAF = 0.5%) and chr4:157997598 (P_TG_ = 6.31 × 10^−08^, LOD_TG_ = 3.13, MAF = 0.5%) were detected with exome-wide significance. These are two rare SNPs with 12 heterozygous and no homozygous carriers. SNP rs189547099 is an intronic variant for both the chromosome 4 open reading frame 45 gene (*C4orf45*) and folliculin interacting protein 2 gene (*FNIP2*). *C4orf45* is an uncharacterized gene with unknown biological function. GTEx^27^ has detected that *C4orf45* is strongly expressed in testis, yet there was almost no expression in other tissues. *FNIP2* is a tumor suppressor gene that has been shown to be involved in regulating the apoptosis signaling pathway in tumors and is responsible for cellular metabolism and nutrient sensing^28,29^. SNP chr4:157997598 is an intronic variant in the glycine receptor beta gene (*GLRB*). This gene encodes the beta subunit of the glycine receptor and has been shown to function as a neurotransmitter-gated ion channel. Mutations in this gene have been shown to cause startle disease^30,31^. Interestingly, SNP chr4:157997598 is located in a CpG island and modifies the binding consensus sequence for a transcription factor zinc finger protein 263 (*ZNF263*) (Figure S6). Although no biological mechanism was found between GLRB and lipid metabolism, it is possible that SNP chr4:157997598 regulates TG levels through *ZNF263*.

Among the 53 variants identified for replication, synonymous SNP rs1160983 in exon 5 of the translocase of outer mitochondrial membrane 40 gene (*TOMM40*) exhibited the strongest association signal after replication and meta-analysis with six independent cohorts (P_LDL_ = 4.44 × 10^−17^, MAF = 2.7%). Before meta-analysis, this variant was nominally associated in IRASFS (P_LDL_ = 8.61 × 10^−06^). *TOMM40* has been shown to be the forming subunit of the translocase of the mitochondrial outer membrane complex and is essential for the import of protein precursors into mitochondria^32^. Genetic studies have identified two adjacent genes (*APOE* and *TOMM40*) in this region to be highly associated with circulating lipid levels^4,33^. After reviewing previously identified *APOE* variants, SNP rs7412 has the highest LD with rs1160983 (r^2^ = 0.49, D′ = 0.94). In IRASFS, rs7412 was nominally associated with LDL (P_LDL_ = 6.61 × 10^−06^). Interestingly, after adjusting for the *APOE* variant (rs7412), rs1160983 remained nominally significant (P = 9.10 × 10^−03^) with LDL. This suggests that the known *APOE* signals do not fully explain the rs1160983 signal in *TOMM40*. It is possible that *TOMM40* may directly contribute to the regulation of LDL levels or SNP rs1160983 may influence *APOE* expression.

An interesting observation from this study is the lack of overlap between the majority of linkage and association signals, even with exome sequencing data. One explanation is that association and linkage capture different mechanisms of phenotypic contributions. Association analysis detects signals that statistically associate with phenotypic variability either directly or through linkage disequilibrium (LD) and thus targets more proximal effects. Linkage detects the co-segregation of an allele with the phenotype in families and therefore can detect long-range effects due to limited recombination events across successive generations. Therefore, each approach has its advantages and limitations. For example, association analysis has gained much success in common variant analysis while often suffering from reduced power to detect rare variants, e.g. statistical power is largely affected by inadequate sample size and limited LD with proximal common variants. In contrast, linkage analysis performance is largely dependent on family structures as well as the number of segregation events, e.g. when family structures are incomplete or allele segregation information is incomplete (only two generations or the parental generation allele information is missing), linkage analysis performance is largely dampened. On the other hand, rare variants (in populations) that are missed by association can be relatively common in a given family with segregation across multiple generations. In this scenario, linkage has increased power over association. Taken together, both association and linkage analysis are valuable approaches for analyzing sequencing data in genetic studies, providing potentially independent information.

Despite multiple strong signals identified, study limitations exist. First, the modest sample size in IRASFS (n = 1,205) limits the power for the association analysis of rare variants, especially given the fact that 82% of the variants analyzed had a MAF < 5%. As a complementary approach, linkage analysis in 90 pedigrees was performed and identified signals that were likely missed by association. Unfortunately, linkage analysis was unavailable in the replication cohorts, and therefore meta-analysis of linkage signals was not performed. Dyslipidemia was not required for participation in IRASFS, thus the majority of individuals were metabolically normal, potentially providing limited enrichment of genetic risk alleles. Third, exome sequencing was not available in replication cohorts, and therefore replication was limited to GWAS imputed or proxy variants only. This approach modestly limited the number of SNPs for replication. Also, while all cohorts were of Mexican ancestry, different ascertainment criteria were used. For example, BetaGene recruited participants at high risk of gestational diabetes while HTN-IR recruited participants at high risk of hypertension. This differs from IRASFS which is a population-based study recruited for large family size.

In summary, exome-wide association and linkage analyses were performed using exome sequencing data in 1,205 Mexican Americans from IRASFS. Multiple signals were detected with circulating lipid levels and top signals were analyzed in six additional independent cohorts for replication. Our results suggested multiple lipid genetic signals in *APOA5*, *TOMM40*, and *GLRB*/*C4orf45*, fine-mapped known lipid genes with exome sequencing data in IRASFS, and explored a combined approach of association and linkage analyzing sequencing data. These results confirm that exome sequencing is a powerful tool to screen for functional genetic variants in the population.

## Methods

### Insulin Resistance Atherosclerosis Family Study (IRASFS)

The study design, recruitment, and phenotyping for the IRASFS has been previously described^34^. In brief, the IRASFS was designed to investigate the genetic and environmental basis of insulin resistance and adiposity. Mexican Americans included in this cohort (n = 1,205 individuals, 90 pedigrees) were recruited from clinical centers in San Antonio, TX and San Luis Valley, CO. Recruitment was based on reported family size and not on health status. Phenotype acquisition and variable calculations have been previously described^34,35^. In brief, TC, TG, and HDL were measured from fasting plasma with standards and LDL was calculated using the Friedewald formula. The study protocol was approved by the Institutional Review Board of each participating clinical and analysis site and all participants provided their written informed consent. All methods in this study were carried out in accordance with the principles of the Declaration of Helsinki.

### Exome Sequencing

Exome sequencing was performed at Texas Biomedical Research Institute using the Illumina Nextera Exome Enrichment System in conjunction with the Illumina HiSeq 2500 sequencer. All sequence reads passed through the Illumina Data Analysis Pipeline, and those from samples passing QC criteria were mapped to the human genome reference sequence (hg19). A detailed description of the sequencing platform and analysis pipeline has been published^36^. Of note, multi-sample recalibration was performed prior to variant calling. The datasets generated and/or analyzed during the current study are available from the corresponding author on reasonable request.

### Statistical Analysis

To ensure normality, high density lipoprotein (HDL), total cholesterol (TC), and triglycerides (TG) were natural log-transformed; low density lipoprotein (LDL) was square root-transformed. While lipid medications were carefully evaluated, the majority of the participants in IRASFS were metabolically healthy with a lipid medication rate of 3.9%. Therefore, only a single regression model was performed without accounting for lipid medication status. Heritability of lipid levels was estimated using Sequential Oligogenic Linkage Analysis Routines (SOLAR)^37^ adjusting for age, sex, body mass index (BMI), and recruitment center. Variants with Mendelian inconsistencies were removed (n = 4,024), resulting in a final number of 548,889 variants^38^. Each variant was coded to an additive model based on the minor allele (reference allele). Genetic models of association were calculated adjusting for age, sex, BMI, recruitment center, and admixture estimates. Admixture estimates were calculated as described previously^39^ using maximum likelihood estimation of individual ancestries as implemented in ADMIXTURE^40^. Tests of association between individual variants and quantitative traits were computed using the Wald test from the variance component model implemented in SOLAR. Burden tests were computed using famSKAT^41^. Gene units were defined using the UCSC gene definition file from NCBI genome build 37 (hg19) whereby each alternatively spliced transcript is included for a total of 39,173 genes. For family-based linkage analysis, variant-specific identity-by-descent (IBD) probabilities were computed using the Monte Carlo method implemented in SOLAR^42^. Two-point linkage was performed using the variance components method implemented in SOLAR, with adjustment of age, gender, BMI, and recruitment center^42^. Variant annotation was performed using ANNOVAR^43^.

### Replication and Meta-analysis

Six cohorts participating in the Genetics Underlying Diabetes in Hispanics (GUARDIAN) Consortium^44^ provided in silico replication data: the Insulin Resistance Atherosclerosis Study (IRAS^45^), BetaGene^46–49^, the Troglitazone in Prevention of Diabetes Study (TRIPOD^50,51^), the Hypertension-Insulin Resistance Family Study (HTN-IR^52,53^), the Mexican-American Coronary Artery Disease Study (MACAD^54–56^) and the NIDDM-Atherosclerosis Study (NIDDM-Athero^57^). All study protocols were approved by the local institutional review committees and all participants gave their informed consent. GWAS genotyping was supported through the GUARDIAN Consortium^44^ using the Illumina OmniExpress array (Illumina Inc.; San Diego, CA, USA) and imputation was performed centrally using IMPUTE2^58^ and the 1000 Genomes phase l integrated reference panel (March 2012). Variants included in analysis had confidence scores > 0.90 and information scores > 0.50. A detailed description of quality control has been described previously^39^.

A total of 56 nominally associated SNPs (P < 5.00 × 10^−05^) with minor allele frequency (MAF) ≥ 1.0% as well as two rare SNPs that reached exome-wide significance (P < 9.11 × 10^−08^, MAF < 1.0%) were selected for replication in the GUARDIAN Consortium. Meta-analysis was performed among IRASFS (n_max_ = 1,205), IRAS (n_max_ = 181), BetaGene (n_max_ = 1,218), TRIPOD (n_max_ = 125), HTN-IR (n_max_ = 763), MACAD (n_max_ = 749), and NIDDM-Athero (n_max_ = 244) using the 1000 Genomes imputation dataset. Overall, 49 SNPs were directly tagged in replication cohorts and four SNPs were tagged by a proxy SNP (r^2^ > 0.6). However, no available proxies were found for the remaining five SNPs, which were excluded, resulting in a total of 53 SNPs in the meta-analysis. The meta-analysis was computed using METAL (http://csg.sph.umich.edu/abecasis/metal). Considering the differential study designs, a weighted meta-analysis of the p-values and samples sizes accounting for direction of effect was performed.

### Previously identified signals

Previously identified lipid loci (N = 157) from the recent GLGC^4^ were extracted and all exome sequencing variants within ± 100 kb of the reported index SNPs were selected for fine-mapping. Conditional association and linkage analyses were performed with the GLGC index SNP as an adjusting covariate.

### ENCODE RNA sequencing data

Four human primary hepatocytes RNA sequencing results were plotted using the UCSC genome browser^24,26^. The four liver biopsy samples included were derived from four European individuals: GSM2072386 (20-week female), GSM2072387 (22-week male), GSM2072372 (32-year male), and GSM2072373 (6-year female).

## Electronic supplementary material

Supplement

Supplementary Dataset
