# Supplementary material for: Exome Sequencing Identifies Genetic Variants Associated with Circulating Lipid Levels in Mexican Americans: The Insulin Resistance Atherosclerosis Family Study (IRASFS)
Source: Sci Rep. 2018 Apr 4;8:5603. doi: 10.1038/s41598-018-23727-2 (PMC5884862; doi:10.1038/s41598-018-23727-2)
Supplement: Supplementary file 1 — Supplement [file 41598_2018_23727_MOESM1_ESM.pdf]

Supplementary Data

Exome Sequencing Identifies Genetic Variants Associated with Circulating Lipid Levels in Mexican Americans: The Insulin Resistance Atherosclerosis Family Study (IRASFS)

Chuan Gao, Keri L. Tabb, Latchezar M. Dimitrov, Kent D. Taylor, Nan Wang, Xiuqing Guo, Jirong Long, Jerome I. Rotter, Richard M. Watanabe, Joanne E. Curran, John Blangero, Carl D. Langefeld, Donald W. Bowden, Nicholette D. Palmer

Supplementary Figure Legends

Figure S1. Distribution of minor allele frequencies among analyzed variants.

Figure S2. Results from the maximum likelihood estimation of individual ancestries as implemented in ADMIXTURE. Unsupervised clustering was conducted for Mexican American participants from IRASFS (gray) with inclusion of representative HapMap samples: CEPH (red), YRI (blue), CHN (green), and MEX (orange).

Figure S3. QQ-plot of results from the association analysis. a. HDL, b. LDL, c. TC, d. TG. Variants included had a minor allele count (MAC) ≥5.

Figure S4. Regional plot of the *APOA5* locus in IRASFS. a) association with TG adjusted for age, sex, BMI, and recruitment center b) association with TG adjusted for age, sex, BMI, recruitment center and rs964184. Genotyped SNPs passing quality control measures are plotted with their Discovery p-values (as -log10 values) as a function of genomic position (hg19). In each panel, the index variant is represented by a purple diamond. Color of additional variants indicates correlation with the index SNP (red, r2≥0.80; orange, 0.60≤r2<0.80; green, 0.40≤r2<0.60; blue, 0.20≤r2<0.40; white, r2<0.20; gray no r2 value available) based on pairwise r^2 values from HapMap. Estimated recombination rates (taken from HapMap) are plotted to reflect the local LD structure. Gene annotations were taken from the University of California Santa Cruz genome browser.

Figure S5. Location of rs2072560 in *APOA5* among ENCODE data. Four human primary hepatocytes RNA sequencing results were plotted using the UCSC genome browser. The four liver biopsy samples included were derived from four European individuals: GSM2072386 (20-week female), GSM2072387 (22-week male), GSM2072372 (32-year male), and GSM2072373 (6-year female).

Figure S6. Location of SNP chr4:157997598 within a ZNF263 binding site. ENCODE ChIP-seq results are plotted using the UCSC genome browser.

Supplementary Table Legends (Tables attached as .xlsx file)

Table S1. Heritability estimates for lipid traits in the IRASFS cohort.

Table S2. Nominally associated ($P \leq 1.00E-04$) and linked signals ($LOD \geq 2.0$) for lipid traits from the IRASFS cohort.

Table S3. Burden testing among IRASFS WES data with lipid traits.

Table S4. SNPs with a LOD score of greater than or equal to 3 with lipid traits in IRASFS.

Table S5. Meta-analysis results for 58 SNPs evaluated for replication with lipid traits.

Table S6. Fine-mapping of previously reported lipid loci in IRASFS conditioned on the index variant.

Figure S1.

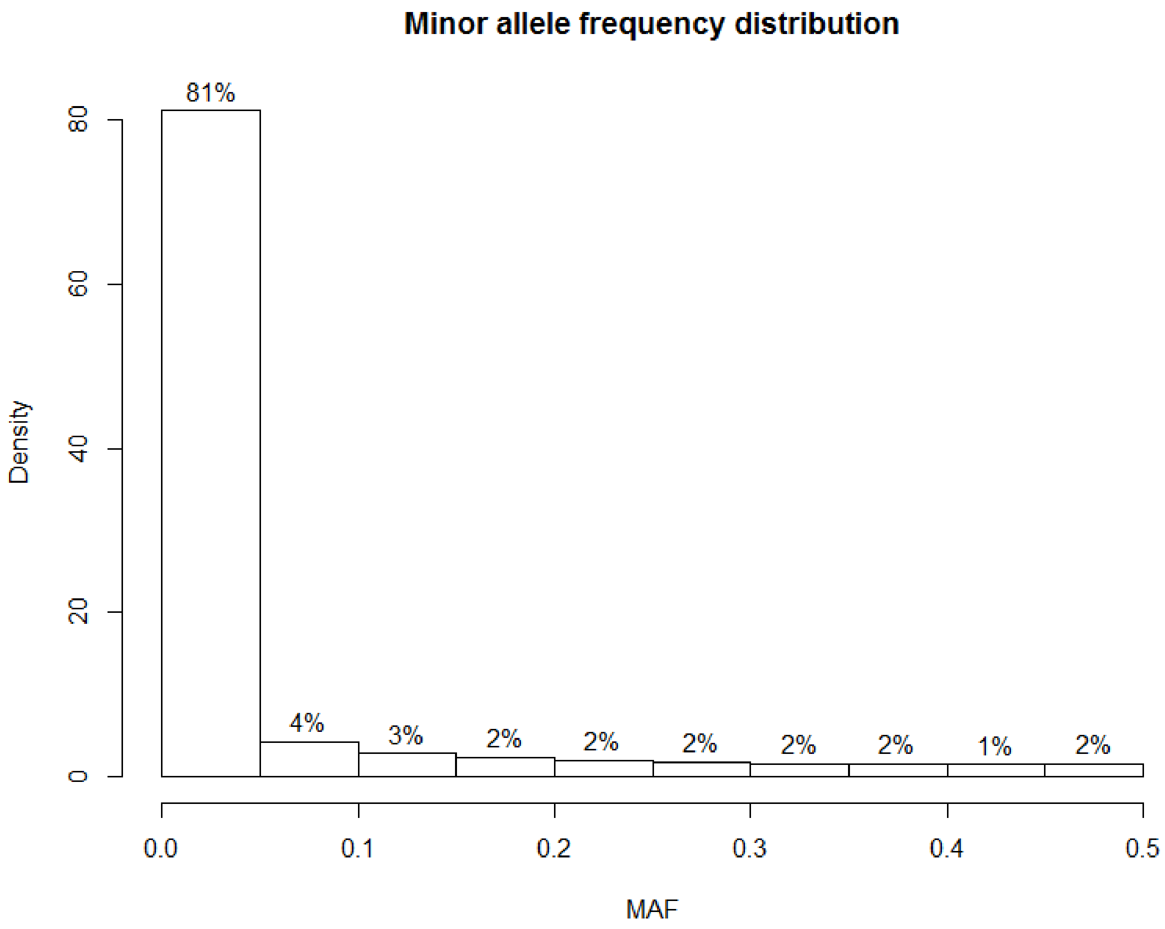
Minor allele frequency distribution
81%
4%
3%
2%
2%
2%
2%
2%
1%
2%
Density
0
20
40
60
80
0.0
0.1
0.2
0.3
0.4
0.5
MAF

Figure S2.

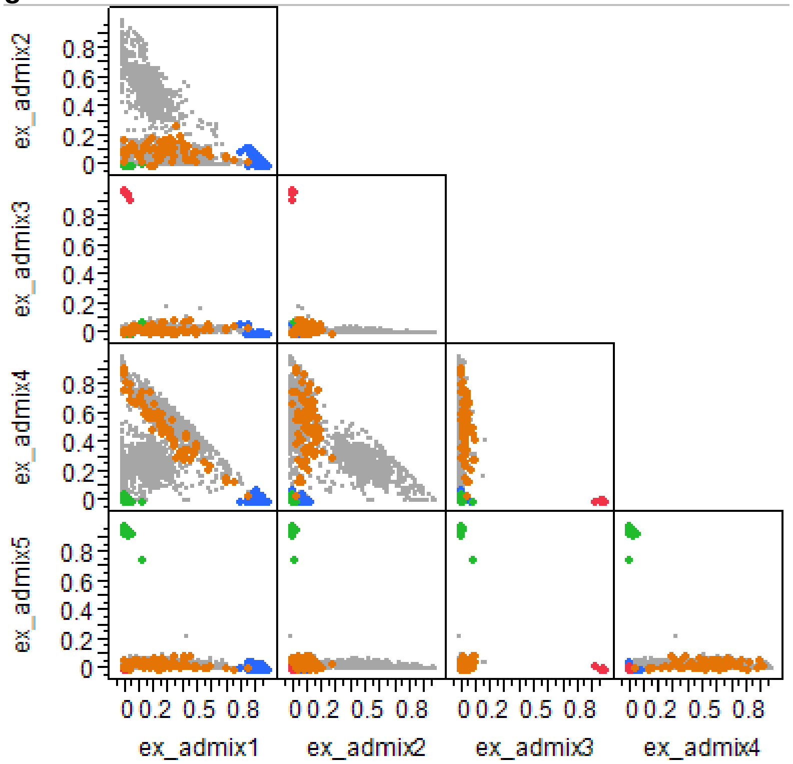
ex_admix2
ex_admix3
ex_admix4
ex_admix5
0
0.2
0.4
0.6
0.8
0 0.2 0.5 0.8
ex_admix1
ex_admix2
ex_admix3
ex_admix4

Figure S3.

A.

Q-Q plot for loghdl p-values

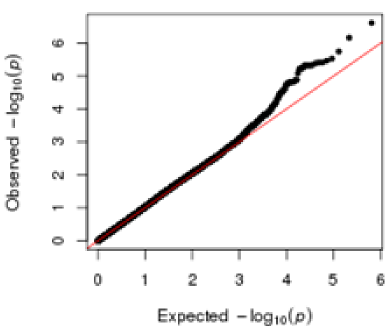

B.

Q-Q plot for sqrtldl p-values

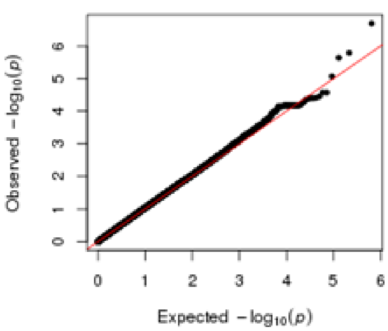

C.

Q-Q plot for logchol p-values

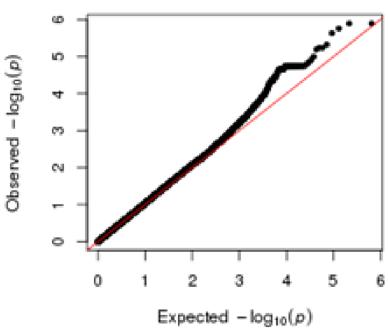

D.

Q-Q plot for logtrigs p-values

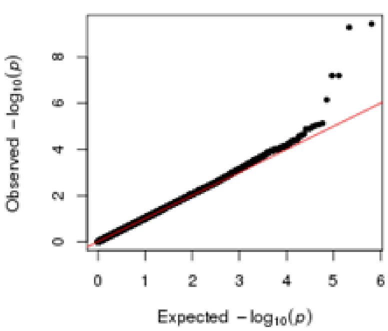

Figure S4.

A.

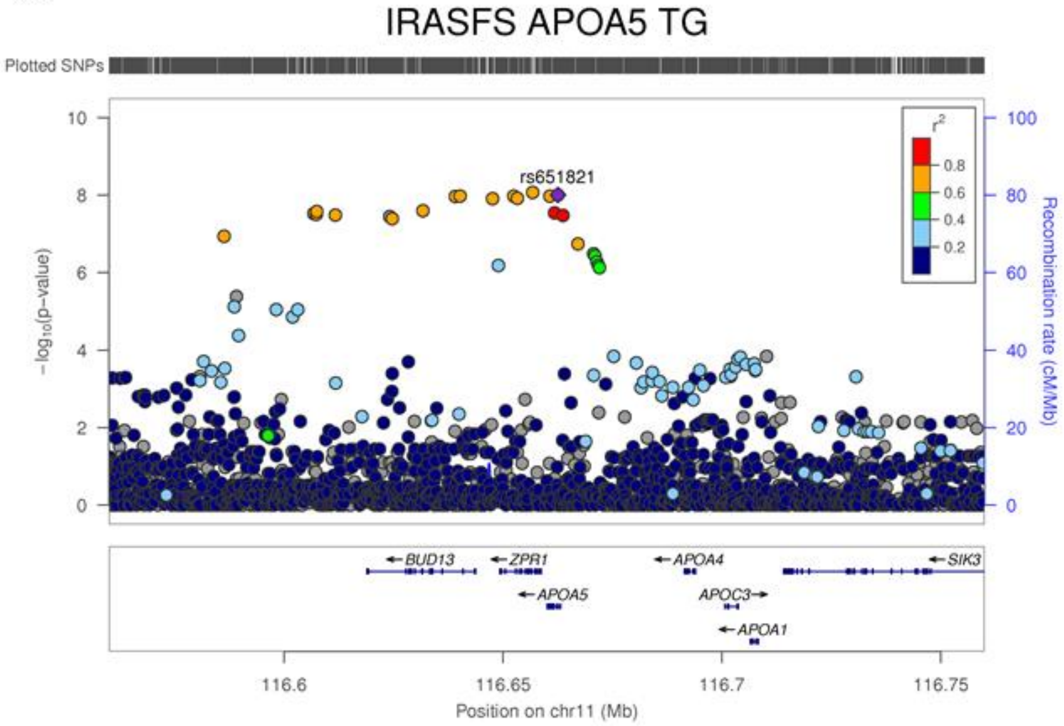

IRASFS APOA5 TG
Plotted SNPs
rs651821
−log10(p−value)
Recombination rate (cM/Mb)
r2
0.8
0.6
0.4
0.2
BUD13
ZPR1
APOA5
APOA4
APOC3
APOA1
SIK3
116.6
116.65
116.7
116.75
Position on chr11 (Mb)

B.

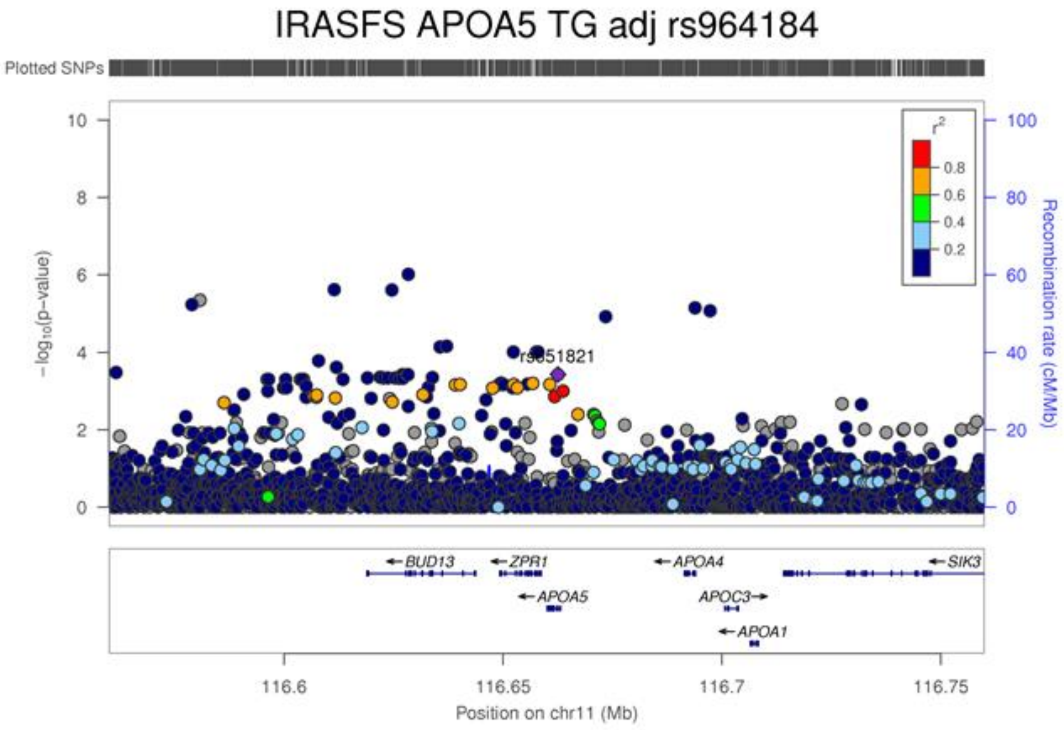

IRASFS APOA5 TG adj rs964184
Plotted SNPs
rs651821
−log10(p−value)
Recombination rate (cM/Mb)
r2
0.8
0.6
0.4
0.2
BUD13
ZPR1
APOA5
APOA4
APOC3
APOA1
SIK3
116.6
116.65
116.7
116.75
Position on chr11 (Mb)

Figure S5.

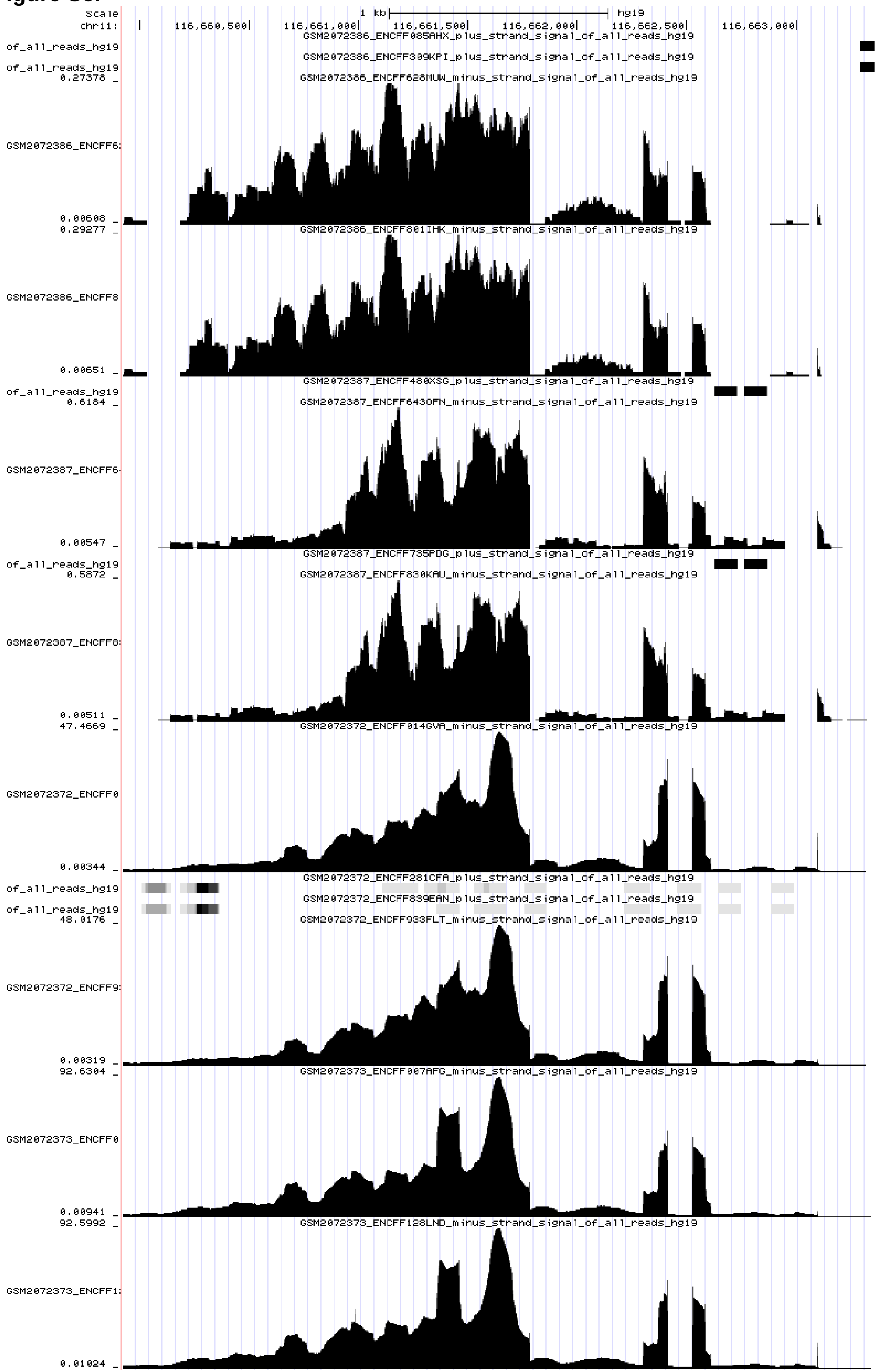

Scale
1 kb
hg19
chr11:
116,660,500
116,661,000
116,661,500
116,662,000
116,662,500
116,663,000
GSM2072386_ENCFF085AHX_plus_strand_signal_of_all_reads_hg19
GSM2072386_ENCFF309KPI_plus_strand_signal_of_all_reads_hg19
GSM2072386_ENCFF628MUW_minus_strand_signal_of_all_reads_hg19
GSM2072386_ENCFF801IHK_minus_strand_signal_of_all_reads_hg19
GSM2072387_ENCFF480XSG_plus_strand_signal_of_all_reads_hg19
GSM2072387_ENCFF643OFN_minus_strand_signal_of_all_reads_hg19
GSM2072387_ENCFF735PDG_plus_strand_signal_of_all_reads_hg19
GSM2072387_ENCFF830KAU_minus_strand_signal_of_all_reads_hg19
GSM2072372_ENCFF014GVA_minus_strand_signal_of_all_reads_hg19
GSM2072372_ENCFF281CFA_plus_strand_signal_of_all_reads_hg19
GSM2072372_ENCFF839EAN_plus_strand_signal_of_all_reads_hg19
GSM2072372_ENCFF933FLT_minus_strand_signal_of_all_reads_hg19
GSM2072373_ENCFF007AFG_minus_strand_signal_of_all_reads_hg19
GSM2072373_ENCFF128LND_minus_strand_signal_of_all_reads_hg19
of_all_reads_hg19
of_all_reads_hg19
0.27376
GSM2072386_ENCFF6:
0.00608
0.29277
GSM2072386_ENCFF8
0.00651
of_all_reads_hg19
0.6184
GSM2072387_ENCFF6·
0.00547
of_all_reads_hg19
0.5872
GSM2072387_ENCFF8:
0.00511
47.4669
GSM2072372_ENCFF0
0.00344
of_all_reads_hg19
of_all_reads_hg19
48.0176
GSM2072372_ENCFF9:
0.00319
92.6304
GSM2072373_ENCFF0
0.00941
92.5992
GSM2072373_ENCFF1:
0.01024

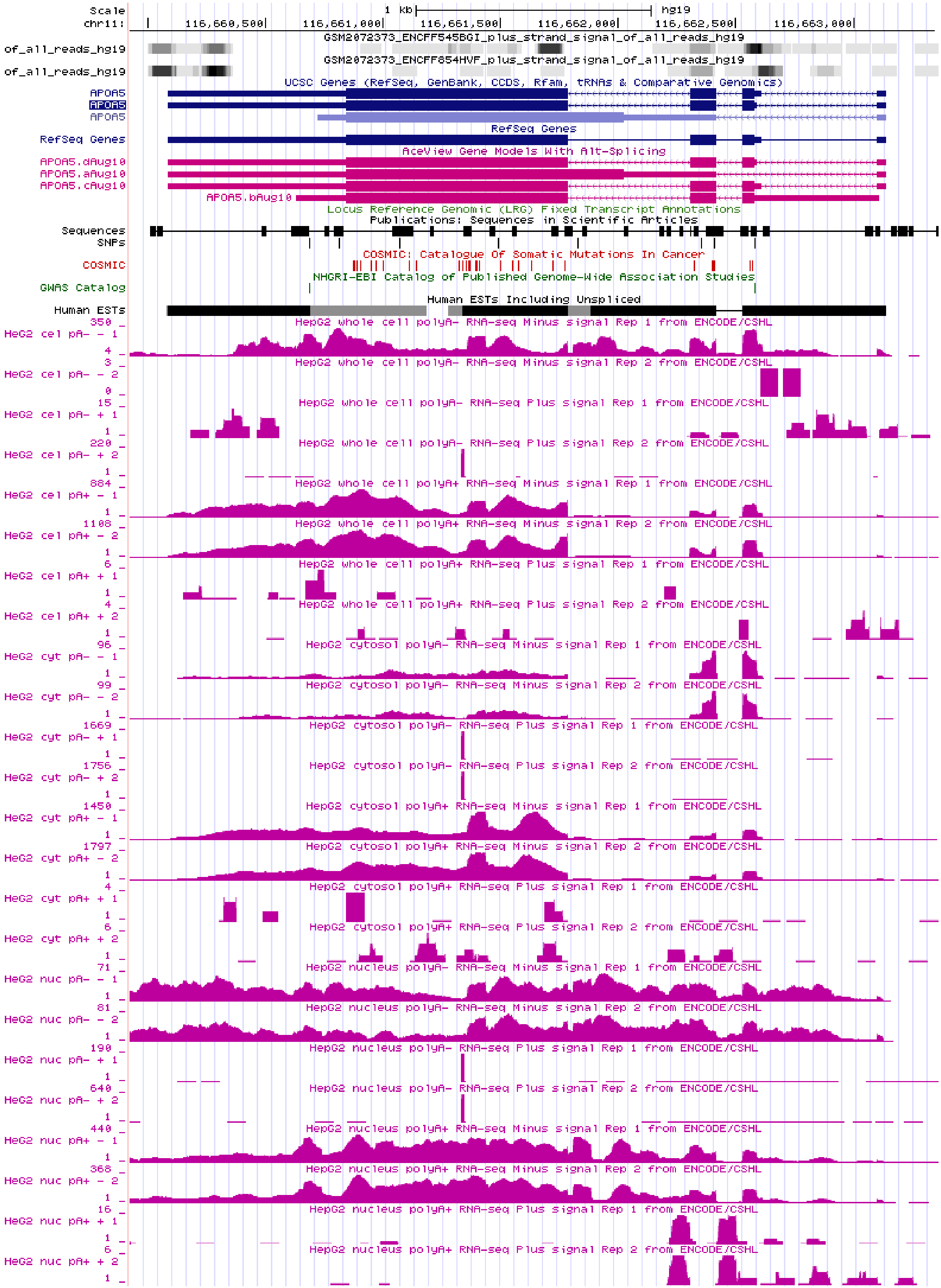

Scale
chr11:
1 kb
hg19
116,660,500
116,661,000
116,661,500
116,662,000
116,662,500
116,663,000
GSM2072373_ENCFF545BGI_plus_strand_signal_of_all_reads_hg19
GSM2072373_ENCFF854HVF_plus_strand_signal_of_all_reads_hg19
of_all_reads_hg19
of_all_reads_hg19
UCSC Genes (RefSeq, GenBank, CCDS, Rfam, tRNAs & Comparative Genomics)
APOA5
APOA5
APOA5
RefSeq Genes
RefSeq Genes
AceView Gene Models With Alt-Splicing
APOA5.dAug10
APOA5.aAug10
APOA5.cAug10
APOA5.bAug10
Locus Reference Genomic (LRG) Fixed Transcript Annotations
Publications: Sequences in Scientific Articles
Sequences
SNPs
COSMIC: Catalogue Of Somatic Mutations In Cancer
COSMIC
NHGRI-EBI Catalog of Published Genome-Wide Association Studies
GWAS Catalog
Human ESTs Including Unspliced
Human ESTs
HepG2 whole cell polyA- RNA-seq Minus signal Rep 1 from ENCODE/CSHL
HepG2 whole cell polyA- RNA-seq Minus signal Rep 2 from ENCODE/CSHL
HepG2 whole cell polyA- RNA-seq Plus signal Rep 1 from ENCODE/CSHL
HepG2 whole cell polyA- RNA-seq Plus signal Rep 2 from ENCODE/CSHL
HepG2 whole cell polyA+ RNA-seq Minus signal Rep 1 from ENCODE/CSHL
HepG2 whole cell polyA+ RNA-seq Minus signal Rep 2 from ENCODE/CSHL
HepG2 whole cell polyA+ RNA-seq Plus signal Rep 1 from ENCODE/CSHL
HepG2 whole cell polyA+ RNA-seq Plus signal Rep 2 from ENCODE/CSHL
HepG2 cytosol polyA- RNA-seq Minus signal Rep 1 from ENCODE/CSHL
HepG2 cytosol polyA- RNA-seq Minus signal Rep 2 from ENCODE/CSHL
HepG2 cytosol polyA- RNA-seq Plus signal Rep 1 from ENCODE/CSHL
HepG2 cytosol polyA- RNA-seq Plus signal Rep 2 from ENCODE/CSHL
HepG2 cytosol polyA+ RNA-seq Minus signal Rep 1 from ENCODE/CSHL
HepG2 cytosol polyA+ RNA-seq Minus signal Rep 2 from ENCODE/CSHL
HepG2 cytosol polyA+ RNA-seq Plus signal Rep 1 from ENCODE/CSHL
HepG2 cytosol polyA+ RNA-seq Plus signal Rep 2 from ENCODE/CSHL
HepG2 nucleus polyA- RNA-seq Minus signal Rep 1 from ENCODE/CSHL
HepG2 nucleus polyA- RNA-seq Minus signal Rep 2 from ENCODE/CSHL
HepG2 nucleus polyA- RNA-seq Plus signal Rep 1 from ENCODE/CSHL
HepG2 nucleus polyA- RNA-seq Plus signal Rep 2 from ENCODE/CSHL
HepG2 nucleus polyA+ RNA-seq Minus signal Rep 1 from ENCODE/CSHL
HepG2 nucleus polyA+ RNA-seq Minus signal Rep 2 from ENCODE/CSHL
HepG2 nucleus polyA+ RNA-seq Plus signal Rep 1 from ENCODE/CSHL
HepG2 nucleus polyA+ RNA-seq Plus signal Rep 2 from ENCODE/CSHL
HeG2 cel pA- - 1
HeG2 cel pA- - 2
HeG2 cel pA- + 1
HeG2 cel pA- + 2
HeG2 cel pA+ - 1
HeG2 cel pA+ - 2
HeG2 cel pA+ + 1
HeG2 cel pA+ + 2
HeG2 cyt pA- - 1
HeG2 cyt pA- - 2
HeG2 cyt pA- + 1
HeG2 cyt pA- + 2
HeG2 cyt pA+ - 1
HeG2 cyt pA+ - 2
HeG2 cyt pA+ + 1
HeG2 cyt pA+ + 2
HeG2 nuc pA- - 1
HeG2 nuc pA- - 2
HeG2 nuc pA- + 1
HeG2 nuc pA- + 2
HeG2 nuc pA+ - 1
HeG2 nuc pA+ - 2
HeG2 nuc pA+ + 1
HeG2 nuc pA+ + 2

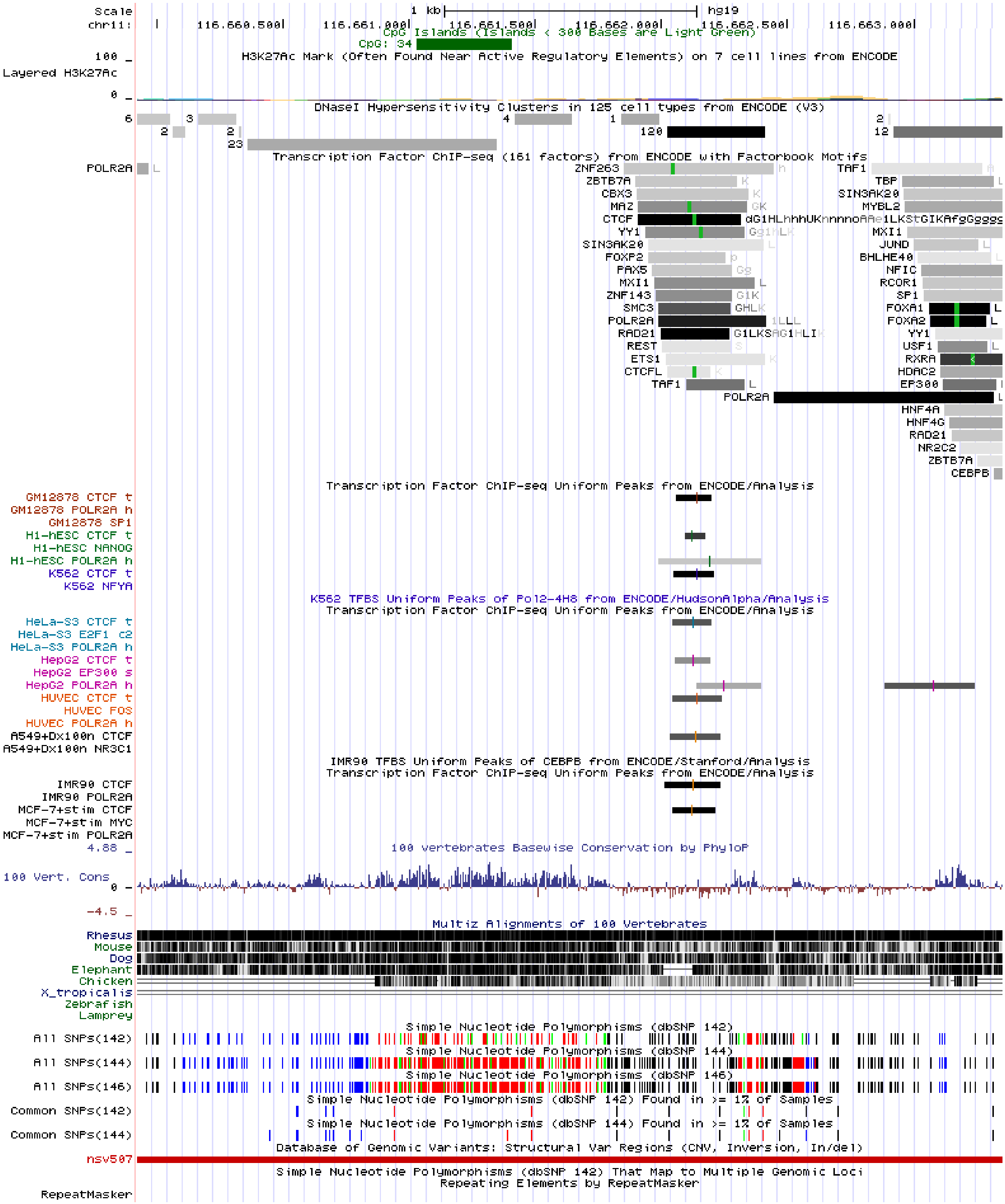

Scale
chr11:
1 kb
hg19
116,660,500
116,661,000
116,661,500
116,662,000
116,662,500
116,663,000
CpG Islands (Islands < 300 Bases are Light Green)
CpG: 34
H3K27Ac Mark (Often Found Near Active Regulatory Elements) on 7 cell lines from ENCODE
Layered H3K27Ac
DNaseI Hypersensitivity Clusters in 125 cell types from ENCODE (V3)
Transcription Factor ChIP-seq (161 factors) from ENCODE with Factorbook Motifs
POLR2A
ZNF263
ZBTB7A
CBX3
MAZ
CTCF
YY1
SIN3AK20
FOXP2
PAX5
MXI1
ZNF143
SMC3
POLR2A
RAD21
REST
ETS1
CTCFL
TAF1
POLR2A
TAF1
TBP
SIN3AK20
MYBL2
MXI1
JUND
BHLHE40
NFIC
RCOR1
SP1
FOXA1
FOXA2
YY1
USF1
RXRA
HDAC2
EP300
HNF4A
HNF4G
RAD21
NR2C2
ZBTB7A
CEBPB
Transcription Factor ChIP-seq Uniform Peaks from ENCODE/Analysis
GM12878 CTCF t
GM12878 POLR2A h
GM12878 SP1
H1-hESC CTCF t
H1-hESC NANOG
H1-hESC POLR2A h
K562 CTCF t
K562 NFYA
K562 TFBS Uniform Peaks of Pol2-4H8 from ENCODE/HudsonAlpha/Analysis
Transcription Factor ChIP-seq Uniform Peaks from ENCODE/Analysis
HeLa-S3 CTCF t
HeLa-S3 E2F1 c2
HeLa-S3 POLR2A h
HepG2 CTCF t
HepG2 EP300 s
HepG2 POLR2A h
HUVEC CTCF t
HUVEC FOS
HUVEC POLR2A h
A549+Dx100n CTCF
A549+Dx100n NR3C1
IMR90 TFBS Uniform Peaks of CEBPB from ENCODE/Stanford/Analysis
Transcription Factor ChIP-seq Uniform Peaks from ENCODE/Analysis
IMR90 CTCF
IMR90 POLR2A
MCF-7+stim CTCF
MCF-7+stim MYC
MCF-7+stim POLR2A
100 vertebrates Basewise Conservation by PhyloP
100 Vert. Cons
4.88
0
-4.5
Multiz Alignments of 100 Vertebrates
Rhesus
Mouse
Dog
Elephant
Chicken
X_tropicalis
Zebrafish
Lamprey
Simple Nucleotide Polymorphisms (dbSNP 142)
All SNPs(142)
Simple Nucleotide Polymorphisms (dbSNP 144)
All SNPs(144)
Simple Nucleotide Polymorphisms (dbSNP 146)
All SNPs(146)
Simple Nucleotide Polymorphisms (dbSNP 142) Found in >= 1% of Samples
Common SNPs(142)
Simple Nucleotide Polymorphisms (dbSNP 144) Found in >= 1% of Samples
Common SNPs(144)
Database of Genomic Variants: Structural Var Regions (CNV, Inversion, In/del)
nsv507
Simple Nucleotide Polymorphisms (dbSNP 142) That Map to Multiple Genomic Loci
Repeating Elements by RepeatMasker
RepeatMasker

Figure S6.

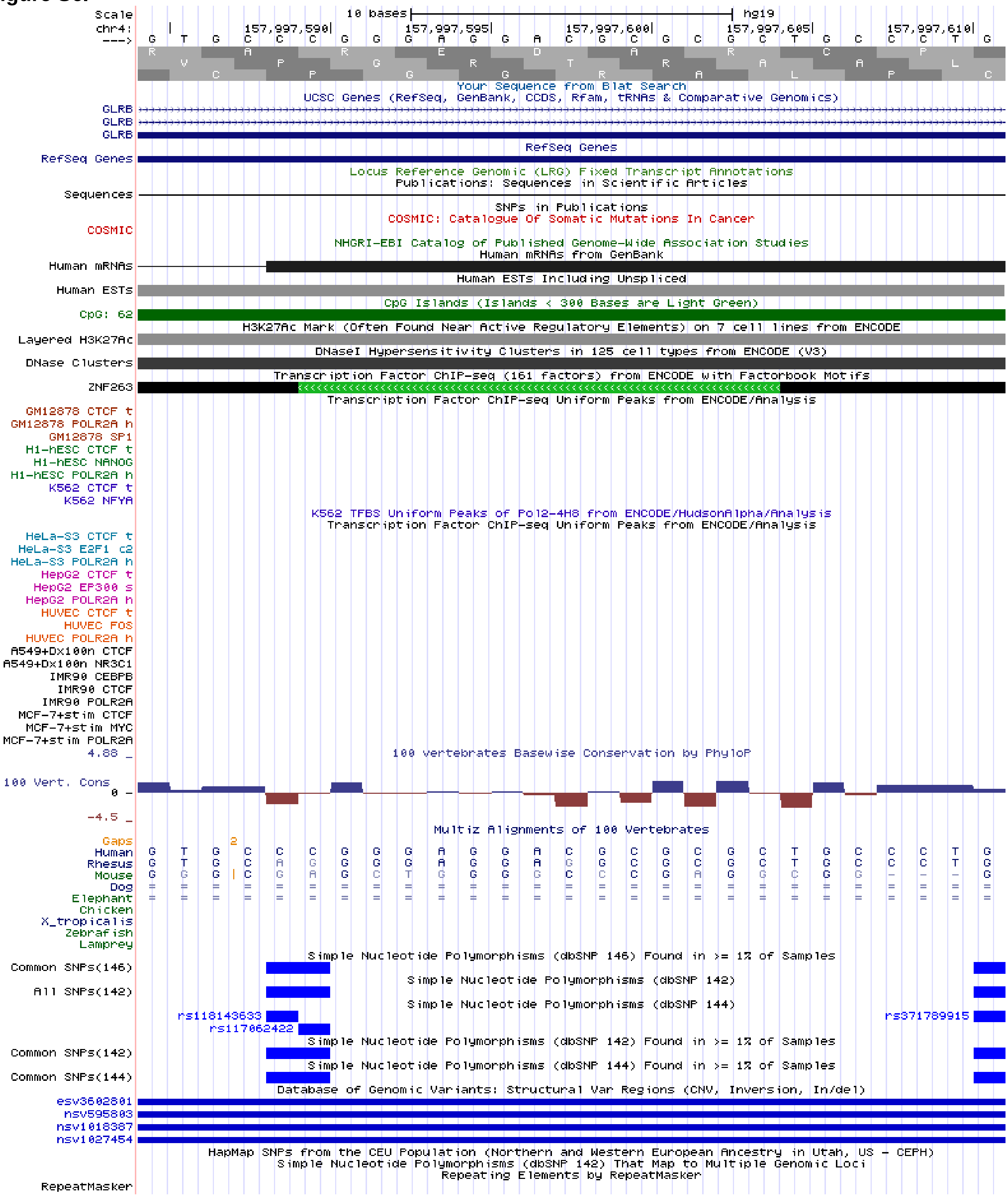

Scale
chr4:
10 bases
hg19
157,997,590
157,997,595
157,997,600
157,997,605
157,997,610
Your Sequence from Blat Search
UCSC Genes (RefSeq, GenBank, CCDS, Rfam, tRNAs & Comparative Genomics)
GLRB
GLRB
GLRB
RefSeq Genes
RefSeq Genes
Locus Reference Genomic (LRG) Fixed Transcript Annotations
Publications: Sequences in Scientific Articles
Sequences
SNPs in Publications
COSMIC: Catalogue Of Somatic Mutations In Cancer
COSMIC
NHGRI-EBI Catalog of Published Genome-Wide Association Studies
Human mRNAs from GenBank
Human mRNAs
Human ESTs Including Unspliced
Human ESTs
CpG Islands (Islands < 300 Bases are Light Green)
CpG: 62
H3K27Ac Mark (Often Found Near Active Regulatory Elements) on 7 cell lines from ENCODE
Layered H3K27Ac
DNaseI Hypersensitivity Clusters in 125 cell types from ENCODE (V3)
DNase Clusters
Transcription Factor ChIP-seq (161 factors) from ENCODE with Factorbook Motifs
ZNF263
Transcription Factor ChIP-seq Uniform Peaks from ENCODE/Analysis
GM12878 CTCF t
GM12878 POLR2A h
GM12878 SP1
H1-hESC CTCF t
H1-hESC NANOG
H1-hESC POLR2A h
K562 CTCF t
K562 NFYA
K562 TFBS Uniform Peaks of Pol2-4H8 from ENCODE/HudsonAlpha/Analysis
Transcription Factor ChIP-seq Uniform Peaks from ENCODE/Analysis
HeLa-S3 CTCF t
HeLa-S3 E2F1 c2
HeLa-S3 POLR2A h
HepG2 CTCF t
HepG2 EP300 s
HepG2 POLR2A h
HUVEC CTCF t
HUVEC FOS
HUVEC POLR2A h
A549+Dx100n CTCF
A549+Dx100n NR3C1
IMR90 CEBPB
IMR90 CTCF
IMR90 POLR2A
MCF-7+stim CTCF
MCF-7+stim MYC
MCF-7+stim POLR2A
100 vertebrates Basewise Conservation by PhyloP
4.88
100 Vert. Cons
0
-4.5
Multiz Alignments of 100 Vertebrates
Gaps
Human
Rhesus
Mouse
Dog
Elephant
Chicken
X_tropicalis
Zebrafish
Lamprey
Simple Nucleotide Polymorphisms (dbSNP 146) Found in >= 1% of Samples
Common SNPs(146)
Simple Nucleotide Polymorphisms (dbSNP 142)
All SNPs(142)
Simple Nucleotide Polymorphisms (dbSNP 144)
rs118143633
rs117062422
rs371789915
Simple Nucleotide Polymorphisms (dbSNP 142) Found in >= 1% of Samples
Common SNPs(142)
Simple Nucleotide Polymorphisms (dbSNP 144) Found in >= 1% of Samples
Common SNPs(144)
Database of Genomic Variants: Structural Var Regions (CNV, Inversion, In/del)
esv3602801
nsv595803
nsv1018387
nsv1027454
HapMap SNPs from the CEU Population (Northern and Western European Ancestry in Utah, US - CEPH)
Simple Nucleotide Polymorphisms (dbSNP 142) That Map to Multiple Genomic Loci
Repeating Elements by RepeatMasker
RepeatMasker
